# A Technology System to Help People With Intellectual Disability and Blindness Find Room Destinations During Indoor Traveling: Case Series Study

**DOI:** 10.2196/65680

**Published:** 2024-11-27

**Authors:** Giulio E Lancioni, Gloria Alberti, Chiara Filippini, Nirbhay N Singh, Mark F O’Reilly, Jeff Sigafoos, Isabella Orlando, Lorenzo Desideri

**Affiliations:** 1 Lega F. D’Oro Research Center Osimo (AN) Italy; 2 College of Medicine Augusta University Augusta, GA United States; 3 Department of Special Education University of Texas at Austin Austin, TX United States; 4 School of Education Victoria University of Wellington Wellington New Zealand; 5 Department of Psychology Sigmund Freud University Milan Italy

**Keywords:** barcode reader, barcode, blindness, intellectual disability, indoor traveling, indoor travel, digital health, travel, navigation, wayfinding, patient care, patient support, mobile health, patient assessment, health intervention, user engagement, technology use, telerehabilitation, rehabilitation, disability, support tools, mobility, orientation, mobile phone

## Abstract

**Background:**

People with severe or profound intellectual disability and visual impairment tend to have serious problems in orientation and mobility and need assistance for their indoor traveling. The use of technology solutions may be critically important to help them curb those problems and achieve a level of independence.

**Objective:**

This study aimed to assess a new technology system to help people with severe to profound intellectual disability and blindness find room destinations during indoor traveling.

**Methods:**

A total of 7 adults were included in the study. The technology system entailed a barcode reader, a series of barcodes marking the room entrances, a smartphone, and a special app that controlled the presentation of different messages (instructions) for the participants. The messages varied depending on whether the participants were (1) in an area between room entrances, (2) in correspondence with a room entrance to bypass, or (3) in correspondence with a room entrance representing the destination to enter. The intervention with the technology system was implemented according to a nonconcurrent multiple baseline design across participants. Sessions included 7 traveling trials, in each of which the participants were to reach and enter a specific room (1 of the 7 or 9 available) to deliver an object they had carried (transported) during their traveling.

**Results:**

The participants’ mean frequency of traveling trials completed correctly was between zero and 2 per session during the baseline (without the system). Their mean frequency increased to between about 6 and nearly 7 per session during the intervention (with the system).

**Conclusions:**

The findings suggest that the new technology system might be a useful support tool for people with severe to profound intellectual disability and blindness.

## Introduction

### Background

People with severe or profound intellectual disability and visual impairment tend to have serious problems in a number of crucial areas, including communication, performance of functional tasks, and orientation and mobility [1-5]. Supporting them in these areas is critically important to (1) promote their developmental process (foster their adaptive behavior and self-determination, and increase their opportunities to live fulfilling life experiences [6-10]) and (2) improve their general condition and, in particular, their well-being and quality of life in full respect of their personal rights [6,7,11-14].

In light of the above, a large variety of intervention programs have been developed, and many of those programs were based on the use of assistive technology, that is, on the use of tools aimed at increasing the possibility of a successful and sustainable outcome, beneficial for the participants and their daily context [15-20]. For example, technology-aided programs have been developed to help people make verbal requests through simple nonverbal responses, such as finger, hand, or toe movements, and to engage in activities requiring the use of objects (eg, collecting and putting away objects) while in a standing or sitting position [19,21-27]).

Technology-aided programs have also been developed to help people manage indoor orientation and traveling and thus improve their level of independence and personal achievement [28-32]. A number of those programs were aimed at guiding the participants from one point to another of a specific (generally small) activity area through the use of sound cues, such as music and verbal encouragements, regulated by electronic control systems [21,32-37]. Other programs used sound cues to guide participants to travel to (reach) specific room destinations with entrances distributed on the sides of a long corridor [38,39]). Their traveling was typically combined with transporting (carrying) objects to those room destinations and thus represented a functional form of activity, which was largely appreciated within the participants’ rehabilitation and care contexts [38,39].

For example, Lancioni et al [39] set up a program of the latter type using (1) a smartphone, which was fixed at the participants’ ankle, and (2) battery-powered light sources, which were displayed before the rooms that the participants were to reach and enter. The smartphone was fitted with audio files, which involved verbal instruction cues such as “Walk,” “Stop,” and “Enter,” as well as preferred stimuli such as music and songs.

At the start of each traveling trial, the participants were accompanied to a departure point (on the side of the corridor on which the room destination was located) and received an object to be transported. Then, the research assistant activated the smartphone, which emitted the verbal cue “Walk” at intervals of 1-2 seconds until the participants reached the entrance of the target room. Such an entrance could be the first, second, or third room the participants encountered while traveling. Once the participants reached the target room, the smartphone’s light sensor was activated by the light source available before the entrance to that room. This led the smartphone to present the instruction “Stop” followed by the instruction “Enter,” which was repeated until the participants entered the room and met a staff member who took the object they had transported, deactivated the smartphone’s instruction function, and activated the smartphone’s stimulation delivery. Once the stimulation was over, the same staff member started a new traveling trial; that is, they accompanied the participants to a new departure point, provided a new object to be transported, and set the smartphone’s instruction function on. The same process was followed for all traveling trials. The results were highly encouraging. Each of the 9 participants showed a large increase in the frequency of traveling trials completed correctly.

### Objectives

The aim of this study was to extend the evidence provided by the Lancioni et al [39] study summarized above through the use of a new technology system. This system presented 2 main (apparently advantageous) differences compared with the previous one. First, it did not rely on light sources activating a smartphone, as these need to be switched on before their use and off thereafter and may represent an obstacle or interference for other people walking in the setting. Rather, it used a barcode reader and different barcode series reproduced on A4 sheets of paper, which were attached to corridor walls before and after the entrances of the rooms included in the setting (sheets of paper that do not need any specific preparation for the sessions and do not constitute a physical obstacle to others sharing the setting with the participants). Second, the new system assisted the participants with a more specific level of instructions during their traveling (details in the *Methods* section). A total of 7 participants with severe to profound intellectual disability and blindness were involved in the study.

## Methods

### Participants

The participants were 6 men and 1 woman, who represented a convenience sample [40], shared the same difficulties in orienting and traveling in indoor areas (eg, in finding room destinations, see below), and attended rehabilitation and care centers. Table 1 lists them by their pseudonyms and reports their chronological age and their age equivalents for receptive communication and daily living skills as measured by the second edition of the Vineland Adaptive Behaviors Scales [41,42]. As shown in Table 1, their chronological age ranged from 25 to 51 years. Their Vineland age equivalents varied between 1 year and 7 months and 3 years and 6 months on receptive communication and between 1 year and 7 months and 4 years on daily living skills (personal subdomain). All participants were diagnosed with severe to profound intellectual disability and blindness and required extensive levels of support from staff and caregivers. Aaron was also unable to walk and relied on the use of a wheelchair. The diagnosis of severe to profound intellectual disability was provided by the psychological services of the centers the participants attended. No IQ scores were available for them.

**Table 1:** Participants’ chronological age and Vineland age equivalents for receptive communication and daily living skills (personal subdomain).

Participants (pseudonyms)	Chronological age (years)	Vineland age equivalents^a^ (years, months)	
		RC^b^	DLSP^c^
Miles	44	1, 7	2, 4
Carter	51	1, 11	2, 5
Noah	25	2, 2	3, 0
Aaron	47	2, 0	1, 7
Colton	40	2, 10	2, 8
Evan	26	1, 7	2, 9
Faith	38	3, 8	4, 0

^a^Age equivalents are based on the Italian standardization of the Vineland scales [41].

^b^RC: receptive communication.

^c^DLSP: daily living skills (personal subdomain).

Their recruitment for the study was based on a number of general conditions. First, all participants had spatial orientation problems that led them to be dependent on their indoor traveling and to remain sedentary and isolated when staff or caregivers were not available to assist them. Second, they had the motor skills necessary to ambulate along corridors and reach different rooms within the centers they attended, with the exception of Aaron. However, Aaron could still manage small-distance indoor traveling using a wheelchair. Third, they were capable of understanding simple verbal instructions such as “Walk,” “Touch the handrail,” “Open the door,” and “Enter.” Fourth, they were known to enjoy a variety of environmental stimuli, and the assumption was that such stimuli could be used as motivating (reinforcing) events at the conclusion of each traveling trial (as participants reached a scheduled room destination) [43,44]. Fifth, staff considered the use of technology to support the participants’ functional traveling engagement a valuable initiative.

### Setting, Traveling Trials, Sessions, Research Assistants, and Stimuli

The setting was represented by corridors and connected rooms (ie, indoor spaces). Specifically, a corridor with room entrances available on both sides was used within each of the centers attended by the participants. A total of 7 or 9 rooms (depending on availability) were used as destinations for the participants’ traveling trials. A traveling trial required the participants to walk (move with the wheelchair) to a specific room destination and enter the room with the object (eg, cup, bottle, or box) they had transported (carried with them). The participants started walking (or moving with the wheelchair in the case of Aaron) from a specific position in the corridor and were to reach and enter the target room, which could be the first, second, or third room they encountered along the way (details in the *Baseline* section). A session included 7 traveling trials, which required the participants to cover a total distance of about 70 to 90 meters. Sessions could occur 1 or 2 times per day, 3 to 6 days a week. The research assistants were 4 women who held a university degree in psychology, had experience using technology-aided programs with people with intellectual and multiple disabilities, and were familiar with data recording procedures.

A variety of music and song stimuli combined with verbal approval were used at the end of each traveling trial (ie, as the research assistant took the object the participant had carried during their traveling). The stimuli were selected following a stimulus preference screening procedure. This procedure involved the presentation of two or three 10-second segments of each song as well as the presentation of each verbal approval expression for a minimum of 10 nonconsecutive times distributed over different screening periods. Selection occurred if the research assistants carrying out the screening agreed that the participants had positive reactions in at least 50% of the stimulus presentations [25].

### Technology System

The technology system included a barcode reader, a series of 10 barcodes, a smartphone, and a special app that controlled the smartphone’s delivery of different types of messages (instructions) for the participants. A mini speaker and headphones were also available. The barcode reader was a commercial device (NETUM Bluetooth 2D Barcode Scanner available on Amazon) that was fixed at the participants’ ankle or waist or attached to Aaron’s wheelchair. It weighed 55 grams, was in a continuous scanning mode, and was linked to the smartphone through Bluetooth. The 10 barcodes were generated through a free online barcode generator. A total of 7 or 9 of those barcodes were used to indicate the 7 or 9 room destinations available for the sessions.

**Figure 1:**
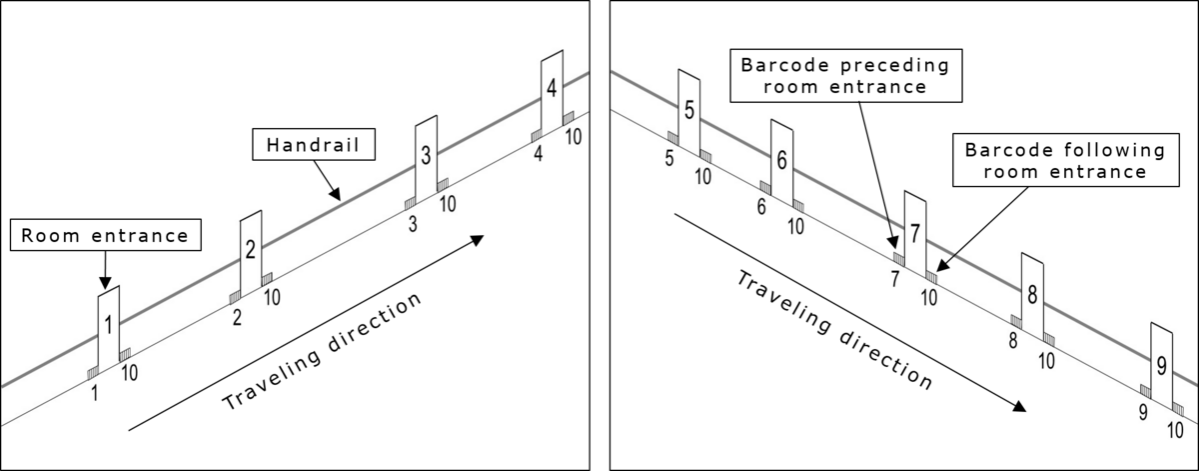
Schematic representation of a corridor with 9 room entrances marked with the numerals 1-9. Each room entrance is preceded by sheets of paper with the barcode for that specific room (see small gray squares marked with the same numeral as the corresponding room entrance). Each room entrance is followed by sheets of paper with the barcode 10. The sheets of paper are placed close to the floor but could also be higher up on the wall.

Figure 1 provides a schematic representation of a corridor with 9 room destinations (entrances) marked with the numerals 1-9. Each room entrance was preceded by 2 or 3 A4 sheets of paper, which were attached to the wall and reported several reproductions of one particular barcode (1 of the 7 or 9 barcodes available to mark the room entrances being used; see Figure 1). A total of 2 or 3 A4 sheets of paper reporting several reproductions of a tenth barcode (a barcode differing from those signaling the room entrances) were attached right after the room entrances (see Figure 1). The special app, which is freely available [45], was developed by Reactive Native Framework to control the smartphone’s emission of 3 different types of messages. Those messages were tied to the participants’ traveling position and consisted of (1) the word “Walk” delivered at 3-second intervals; (2) combinations of words such as “Walk,” “Touch the handrail,” or “Touch the wall” delivered at intervals of 1-2 seconds; and (3) combinations of words such as “Enter,” “NAME enter,” or “Open the door” delivered at intervals of 1-2 seconds. The participants received those messages through a Bluetooth mini speaker they had on their chest or Bluetooth headphones (details in the Intervention section). The smartphone was with the research assistants.

For example, if the departure point for a traveling trial was the area of the corridor preceding room entrance 1 and the destination was room entrance 3 (see the representation in Figure 1), the participants received the first type of message (the word “Walk” delivered at 3-second intervals) until they reached the barcode placed before room entrance 1. When they reached that barcode, the participants received the second type of message (words such as “Walk,” “Touch the handrail,” or “Touch the wall” delivered at intervals of 1-2 seconds). When they reached the tenth barcode (ie, had bypassed the entrance to room 1), they received the first type of message again. This continued until they reached the barcode preceding room entrance 2. At that point, they received the second type of message. The first type of message was restored as they bypassed the entrance to room 2 (ie, met the tenth barcode). Once they reached the barcode signaling the entrance to room 3 (their destination), they were provided with the third type of message, with words such as “Enter,” “NAME enter,” or “Open the door,” delivered at intervals of 1-2 seconds. Once they entered the room, the research assistant took the object they had transported (carried with them during traveling) and activated a prearranged stimulation file on the smartphone. This led the smartphone to play 20 seconds of preferred music preceded by approval words.

If the departure point for a traveling trial was the area of the corridor preceding room entrance 5, and the destination was room entrance 6 (see the representation in Figure 1), the participants initially received the first type of message. Once the barcode reader detected the barcode before room entrance 5, the participants received the second type of message. This was replaced by the first type of message when the barcode reader detected the tenth barcode. As the barcode reader detected the barcode signaling room entrance 6, the participants received the third type of message. The conditions in place when the participants entered the destination were as described above. The same procedural conditions were followed for each of the 7 traveling trials available in every session. During each traveling trial, the participants transported an object that was delivered at the destination.

### Experimental Conditions and Data Analysis

A nonconcurrent multiple baseline design across participants was used for the study [46]. Specifically, all participants started with baseline sessions in which the technology system was not in use. Different numbers of baseline sessions were scheduled for the different participants as required by the design. The baseline was followed by an intervention phase with the use of the technology system. Baseline and intervention sessions were implemented by the research assistants. To ensure a high level of accuracy from the research assistants (ie, a high level of procedural fidelity [47]), 2 strategies were adopted. The first strategy consisted of having the research assistants familiarize themselves with the baseline and intervention conditions during 2 practice sessions. The second strategy involved the use of regular feedback on their performance. Feedback was provided by a research supervisor, who had access to video recordings of the sessions, and consisted of informing the research assistants as to whether they were accurate or needed to make changes in the implementation of the procedural conditions.

The participants’ frequency of traveling trials correctly completed was summarized in graphic form. The difference between the baseline and the intervention data was assessed through the use of the percentage of nonoverlapping data (PND) method [48]. This method, which is one of the most immediate and practical tools for the evaluation of single-case research data, served to determine for each participant the percentage of intervention sessions showing a frequency of correct traveling trials higher than the highest baseline value.

### Baseline

The 5 to 10 baseline sessions available for the participants were carried out in corridors with 7 or 9 room entrances (see the *Setting, Traveling Trials, Sessions, Research Assistants, and Stimuli* section and Figure 1). The system was not available. Different familiar objects were attached to the doors of the room destinations (to help participants discriminate those destinations; see below), and the participants were guided to touch some of those objects before the beginning of every session. At the start of each traveling trial, the research assistants (1) guided the participants to a departure point and helped them place their hand on the handrail, facing the direction of the room they were to reach; (2) provided them with an object; and (3) asked them to enter the room with the same object. For example, the participants could be guided to place their left hand on the handrail right after the entrance to room 2 (see Figure 1), provided with a cup, and asked to enter the room with the cup (eg, room 4).

**Figure 2:**
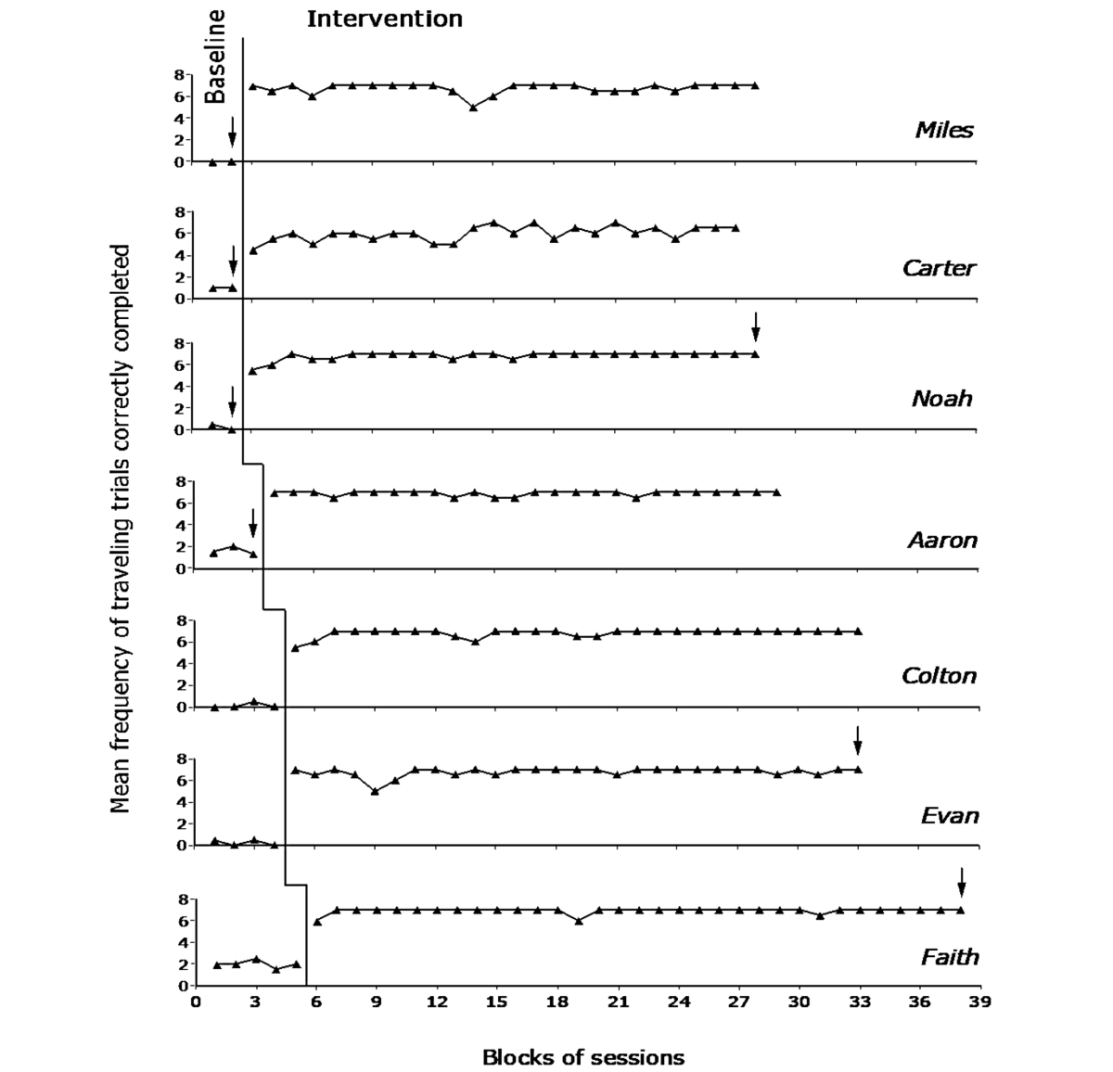
The 7 graphs report the baseline and intervention traveling data for the 7 participants. Each data point represents the mean frequency of traveling trials correctly completed per session over a block of 2 sessions. Blocks of 3 sessions at the end of the baseline or intervention phases are marked with an arrow.

The research assistants intervened with verbal and physical prompts if the participants entered a room preceding the destination (ie, entered room 3), walked past the correct room (ie, continued to walk bypassing the entrance to room 4), or made no progress for about 1 minute. Once the participants entered room 4, the research assistants took the object they had transported during their traveling and provided them with 20 seconds of preferred music preceded by verbal approval. This stimulation was delivered regardless of whether they had completed the traveling trial correctly (independent of research assistants’ prompts) or incorrectly (with research assistants’ prompts). Out of the 7 traveling trials scheduled for each session, 1 or 2 involved reaching and entering the first room on the way (eg, departing from immediately after room 2 and having to reach and enter room 3; see Figure 1), 2 or 3 involved reaching and entering the second room on the way (eg, departing from immediately after room 5 and having to reach and enter room 7; see Figure 1), and 2 or 3 involved reaching and entering the third room on the way (eg, departing from before room 5 and having to reach and enter room 7; see Figure 1). The door of the room to reach and enter always had an object matching the one the participants received from the research assistants at the start of the traveling trial and were to transport to the destination.

### Intervention

During the 50 to 67 intervention sessions, the number and types of traveling trials available per session, the stimulation delivered after the research assistants took the object transported during traveling, and the conditions for research assistants’ prompts were as in the baseline. The crucial difference from the baseline was the use of the technology system (with the consequent removal of the objects attached to the doors of the room destinations). Sheets of paper with the barcodes were used as described in the *Technology System* section. Those sheets were attached to the corridor’s walls before and after the room entrances, either very close to the floor or higher up, depending on whether the participants had the barcode reader fixed at the ankle or at the waist. Aaron had the barcode reader on the wheelchair, so the paper sheets with the barcodes were attached for him higher up on the corridor’s walls.

At the start of each traveling trial, the research assistants (1) guided the participants to a departure point and helped them to place their hand on the handrail, facing the direction of the room door they were to find; (2) provided them with an object; (3) asked them to find and enter the room where to bring that object; and (4) typed the room number in the smartphone. Once the room number was typed in, the smartphone started to deliver the first type of message with the word “Walk” repeated at 3-second intervals. As soon as the participants reached the next door entrance, the smartphone message changed. If the door entrance was the destination, the smartphone delivered the third type of message with words such as “Enter,” “NAME enter,” or “Open the door” delivered at intervals of 1-2 seconds. If the door entrance was not the destination, the smartphone delivered the second type of message with words such as “Walk,” “Touch the handrail,” or “Touch the wall” at intervals of 1-2 seconds. This message continued until the participants had reached the barcode after that entrance, that is, the tenth barcode. Then, the smartphone restarted the delivery of the first message. The smartphone’s delivery of the 3 types of messages always followed the conditions described in the *Technology System* section. When the participants entered the room destination, the research assistants took the object they had transported and activated via the smartphone a 20-second period of preferred music preceded by approval words. The same process was followed for each of the traveling trials included in the session.

During the first 15-20 intervention sessions, the participants received the smartphone messages and preferred stimulation through a Bluetooth mini speaker they had at their chest. Thereafter, they received the messages and preferred stimulation through Bluetooth headphones, so the auditory input was audible only to them, and environmental disturbance was eliminated. The only exception to this was Evan, who continued to use the mini speaker because he had some problems wearing headphones.

The intervention sessions were preceded by 2 to 4 familiarization (practice) sessions. During those sessions, research assistants’ prompts could be frequently used to help the participants respond to the system messages and complete the traveling trials without hesitation or breaks.

### Data Recording

Data recording concerned the traveling trials occurring during the sessions and whether those trials were correctly or incorrectly completed (ie, the participants reached and entered the right rooms independent of research assistants’ prompts or with research assistants’ prompts). The recording also included the time required to complete each trial. Data recording was carried out by the research assistants. Interrater agreement was assessed in at least 23% of the sessions of each participant by having a reliability observer record the data from videos of the sessions. The percentage of agreement (computed by dividing the number of sessions in which the research assistant and reliability observer reported the same number of correct trials and times for the single trials that did not differ more than 20 seconds by the number of sessions involving the presence of the reliability observer and multiplying by 100%) was within the 90% to 100% range for all participants.

### Ethical Considerations

Staff members viewed the participants’ involvement in the study as a positive opportunity for engaging in functional activity involving physical exercise, social interaction, and contingent positive stimulation. The participants’ legal representatives strongly agreed with this view and signed a formal consent authorizing the participants to be included in the study.

The study was approved by the Ethics Committee of the Lega F. D’Oro, Osimo (AN), Italy (approval P030820242). All procedures performed were in accordance with the ethical standards of the institutional and/or national research committee and with the 1964 Helsinki Declaration and its later amendments or comparable ethical standards. Participants were de-identified as pseudonyms were used for them. No compensation was considered or provided for the participants.

## Results

The 7 graphs of Figure 2 report the baseline and intervention traveling data for the 7 participants. Each data point represents the mean frequency of traveling trials correctly completed per session over a block of 2 sessions. Blocks of 3 sessions (which could occur at the end of the baseline or intervention phases) are marked with an arrow. The graphs do not report the 2 to 4 familiarization (practice) sessions preceding the start of the intervention phase.

During the baseline phase, the mean frequency of traveling trials correctly completed per session varied between zero (Miles) and 2 (Faith). Traveling trials correctly completed were mostly those in which the participants were to reach the room entrance nearest (next) to the traveling departure point. The participants did not seem to pay attention to (search and inspect) the objects attached to the doors to identify the rooms they were to enter. The mean time length for the single baseline traveling trials ranged between about 0.5 minutes (Faith) and 2.5 minutes (Aaron), with a mean across participants of 1.3 minutes.

During the intervention phase, the mean frequency of traveling trials correctly completed per session ranged from about 6 (Carter) to nearly 7 (Faith). The PND method produced indices of 1 for all participants. These indices (1) demonstrate that the frequency of traveling trials correctly completed of all the intervention sessions was higher than the highest baseline value, and thus (2) confirm the positive impact of the intervention with the technology system.

The time lengths for the intervention traveling trials were similar to those recorded during the baseline both in terms of ranges and mean across participants. Variations were largely due to participants’ differences in motor and traveling characteristics. For example, shorter trial times were recorded for Faith and Evan, whose ambulation tended to be fairly confident and relatively fast. Longer trial times were recorded for Aaron, who was nonambulatory and relied on the use of a wheelchair for his traveling, and for Miles and Colton, whose ambulatory behavior tended to be fairly slow.

## Discussion

### Principal Findings

The results show that the 7 participants with severe to profound intellectual disabilities and blindness (with or without the lack of ambulatory behavior) improved their ability to travel to specific room destinations during the intervention phase of the study. These data, which confirm previous evidence in the area [39], suggest that the new technology system may represent a valid (advantageous) alternative to the one used previously [39]. In light of the above, several considerations may be in order.

First, enabling people with severe to profound intellectual disability and blindness (or serious visual impairment) to travel across different areas of an indoor setting and make their traveling meaningful by transporting objects to the traveling destinations may be considered a relevant rehabilitation target, respectful of the people’s rights [11,12,49]. Indeed, it provides people with the opportunity to engage in physical exercise, manage the use of objects, practice self-determination, and get in touch with others (ie, all aspects that would have a positive impact on the people’s well-being and quality of life [6,7,9,50-52]). Others were represented by the research assistants during this study, but they could involve different staff members in a daily context [39].

Second, such a rehabilitation target may be extremely difficult to achieve unless adequate technology support is available [9,39,53]. While the application of technology support may require some environmental adjustments that cannot always be taken for granted [20,52,53], relying on extensive staff guidance would preclude participants’ independence and would be beyond the resources of many daily contexts [25,49,51]. Using object signals as discrimination cues for the different room destinations (ie, applying a procedure similar to that available during the baseline) may not be sufficient to obtain satisfactory results.

Third, the technology system used in this study presented 2 main differences compared with that used by Lancioni et al [39]. That is, the new system (1) relied on barcodes reproduced on sheets of paper attached to the corridors’ walls (rather than on light sources) and (2) delivered different types of messages to provide the participants with more specific guidance during their traveling. The sheets of paper with barcodes (contrary to the light sources used in the previous study) do not interfere with the free ambulation of other people sharing the participants’ context and do not require any preparation for the sessions. No data were collected to determine whether the use of specific guidance messages (messages changing according to the different phases of the people’s traveling trials, as done in this study) could promote a better performance compared with the use of relatively generic guidance messages. Notwithstanding this lack of evidence, one could still argue that more specific messages (differentiated instructions) may have an advantage, particularly for people with more extensive difficulties.

Fourth, the participants’ general performance stability over the intervention period may be taken to suggest that (1) the support provided by the technology system was basically adequate to guide their traveling, and (2) they continued to be motivated to complete the traveling trials. The motivation might have been (1) connected to the presence of preferred stimulation at the end of each trial and (2) facilitated by the fact that traveling was not provoking anxiety (ie, apprehension and negative feelings, which are frequently associated with failure and frustration) [33,43,44,54,55].

Fifth, the participants’ use of headphones to receive messages concerning their traveling and preferred stimulation at the end of each traveling trial has important practical implications. In fact, it avoids that the participants’ traveling engagement produces disturbance to other people sharing the context and, consequently, does not limit the participants’ traveling opportunities to specific parts of the day (eg, when others are temporarily absent or unlikely to be particularly troubled) [19,56,57].

Sixth, the technology system entails commercially and easily accessible components, such as the barcode reader, the smartphone, and a specially developed app. The app, which is available at no cost, was set up to ensure that (1) the barcode reader’s identification of the barcodes displayed along the travel routes would provide the smartphone with specific signals and (2) the smartphone would respond to those signals with the delivery of specific and timely messages.

### Limitations and Future Research

The 3 main limitations of the study concern the relatively small number of participants, the lack of maintenance and generalization data, and the absence of a social validation of the system and its applicability. To address the first limitation, new (direct or systematic replication) studies with additional participants will be necessary [58-60]. These studies will provide new evidence that could help determine the robustness and reliability of these findings and, thus, the applicability of an intervention program using the reported technology system. To address the second limitation, new studies will need to include (1) a longer data collection period to assess whether correct traveling performance can be maintained over time and (2) the use of different settings to determine whether the same technology system and procedural conditions can be profitably applied across various contexts [44,61]. To address the third limitation, one could involve staff members and caregivers in (1) watching videos of intervention sessions with the system and (2) providing their ratings of the system’s effectiveness, friendliness to the participants, and applicability in daily environments [62-64].

### Conclusions

In conclusion, the results show that the new technology system was useful in helping people with severe to profound intellectual disability and blindness or blindness and lack of ambulatory behavior in traveling to different room destinations within an indoor setting. These results, which corroborate previous evidence on the effectiveness of technological support in promoting successful indoor traveling, appear very encouraging. Even so, caution is required in drawing conclusions about their robustness and general implications, given that the study presented a number of limitations that new research needs to address. New research may also envisage an upgrade of the technology system that could simplify its functioning and improve its applicability across people and settings.

## Abbreviations

PND: percentage of nonoverlapping data

## Data Availability

The datasets generated during and/or analyzed during this study are available from the corresponding author on reasonable request.

The authors developed the app used to control the smartphone messages during the participants’ travel, which is available at no cost [45].
